# Assessment of Custom-Made Acetabular Implants for Complex Revision Total Hip Arthroplasty: A Concise Follow-up, at a Minimum of 5 Years*

**DOI:** 10.2106/JBJS.25.00876

**Published:** 2026-01-16

**Authors:** Anna Di Laura, Johann Henckel, Alister Hart

**Affiliations:** 1 Royal National Orthopaedic Hospital NHS Trust, Stanmore, United Kingdom; 2 Department of Mechanical Engineering, University College London, London, United Kingdom; 3 Institute of Orthopaedics and Musculoskeletal Science, University College London, London, United Kingdom; 4 Cleveland Clinic London, London, United Kingdom

## Abstract

In this study, we evaluated the long-term outcomes of 3D-printed, custom-made acetabular implants (CMAIs), expanding on earlier, mid-term findings. Radiographic assessment focused on implant migration and bone ingrowth, and clinical outcomes included survivorship, complications, the mobility score, and the Oxford Hip Score (OHS). The median follow-up was 7 years (range, 5 to 9 years). Thirty patients of diverse ethnic backgrounds (21 female; median age of 70 years) were included. Implant migration was limited to the first postoperative year (≤5 mm), with negligible displacement thereafter. Bone ingrowth occurred in 27 (90%) of the patients. The 9-year implant survival rate was 96.3%, with 1 re-revision for infection. The median OHS improved from 8 preoperatively (interquartile range [IQR], 4 to 13) to 32 (IQR, 21 to 37) at the previous, mid-term follow-up (p < 0.0001), and then remained essentially the same at the most recent follow-up. Mobility scores improved by a mean of 1.4 points (p < 0.0001). Two dislocations (in 1 patient), 1 transient sciatic nerve palsy, and recurrent infection in 2 patients were recorded. CMAIs provide durable fixation. Constructs stabilize over time, reflecting osseointegration.

**Level of Evidence:**

Therapeutic Level IV. See Instructions for Authors for a complete description of levels of evidence.

In total hip arthroplasty, custom-made acetabular implants (CMAIs) provide tailored solutions for complex defects when conventional implants are inadequate. Their assessment is difficult because of the variability of defect morphology and treatment approaches, and yet existing evidence suggests functional improvement in cases of severe bone loss, particularly Paprosky type-3A and 3B defects^1,2^. Long-term outcome data remain limited.

Early implant migration has been reported after revision procedures. While small displacements may occur before achieving osseous ingrowth, they do not necessarily indicate construct failure^3,4^. Nonetheless, when migration is observed radiographically, closer follow-up is recommended until stabilization and osseointegration are confirmed^5^.

We previously reported the results at a minimum of 3 years after acetabular reconstruction for the management of Paprosky type-3B defects, with or without pelvic discontinuity, between 2016 and 2019^6^. The aim of the current follow-up study was to assess the longer-term outcomes.

## Materials and Methods

### Study Design

This was a single-center, single-surgeon cohort study of patients who underwent acetabular reconstruction with a CMAI for Paprosky type-3B defects (with or without pelvic discontinuity) between May 2016 and January 2020, with a minimum follow-up of 5 years. Consent was obtained from each patient in accordance with local ethical guidelines. All surgical procedures and follow-up evaluations were performed by the senior author (A.H.). The inclusion criteria focused on Paprosky type-3B defects following 1 or more revisions.

### Surgical Planning and Technique

Acetabular bone loss was assessed using an implant-based analysis, whereby the design process began by virtually modeling the implant to fill the defect. Implant geometry, flanges, and screw holes prioritized the restoration of joint biomechanics, including the center of rotation and offsets^6^.

**Fig. 1:**
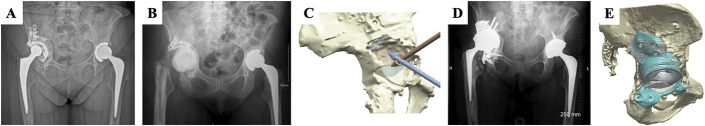
**Figs. 1-A and 1-B:** Anteroposterior pelvic radiographs demonstrating preoperative status with a failed implant (**Fig. 1-A**) and following implant removal (**Fig. 1-B**), before second-stage revision. **Fig. 1-C:** Three-dimensional bone model with simulation of reaming in the “double-bubble” configuration. **Fig. 1-D:** Postoperative radiograph showing restoration of the hip center of rotation. **Fig. 1-E:** Lateral 3D-CT views of the same implant at 1- (gray) and 3-year (blue) postoperative time points. Overlaid scans were used to quantify implant migration, demonstrating good alignment and confirming the absence of migration during follow-up.

Dissection through the gluteal muscles depended on the exposure required. The acetabular cavity was fully exposed and sequentially reamed with hemispherical reamers, and all nonviable bone was excised (to accommodate “bubble” and “double-bubble” constructs) (Fig. 1). Both custom reamers and standard instrumentation were used. No bone grafting was performed. Implant fixation relied on a combination of press-fit and screw fixation, guided by patient-specific drill guides.

All patients were treated with a single-manufacturer, 3D-printed, titanium CMAI (enovis) that incorporated a dual-mobility type of bearing featuring either a 40-mm (50%) or 42-mm (50%) polyethylene liner and a 28-mm head^6-8^. Patients with infection underwent a 2-stage procedure. In cases in which the femoral stem was well fixed, it was retained rather than revised.

### Radiographic Outcome Assessment

Postoperative imaging included radiographs and computed tomography (CT) scans immediately postoperatively, at 6 months, and annually thereafter to assess radiolucency, implant stability, and congruency. Additional imaging was obtained if implant migration was suspected^5^.

Bone ingrowth was assessed radiographically and defined as the presence of trabecular bone extending to the metal surface (“spot welds”). This assessment was performed systematically for all implants on sequential radiographs and CT scans.

CT data were used to compute a relative 3D comparison between component position immediately postoperatively and at longer-term follow-up and to assess the bone-implant contact area^9^. The anterior pelvic plane was used as the standard coordinate system for 3D measurements^10,11^ (Simpleware ScanIP Medical, version 2024.06; Synopsys). Bone-to-bone registration accuracy was assessed using the average surface distance (ASD) between corresponding pelvic models. Nuclear SPECT-CT (single-photon emission computed tomography) was performed when loosening was suspected.

### Clinical Outcome Assessment

Implant survivorship was calculated using the Kaplan-Meier method, with all-cause re-revision as the end point. Failure was defined as revision or replacement of the index implant.

Functional outcomes were assessed using the Oxford Hip Score (OHS)^12^. Some patients had undergone multiple joint reconstructions and had comorbidities that could confound their hip scores. We recorded patients’ level of mobility as follows: 1 (independent walking), 2 (use of 1 cane), 3 (2 canes), 4 (frame and wheeled walker), 5 (elbow crutches), or 6 (wheelchair)^3^.

### Statistical Analysis

Prism (version 10.5.0; GraphPad) was used for statistical analysis. Normality was assessed using a D’Agostino-Pearson test; data were non-normally distributed. A Pearson test was used to assess correlations between the deviation of the center of rotation (CoR) and body mass index (BMI), age, sex, and pelvic discontinuity. Kaplan-Meier curves were used for survival analysis. Significance was set at a p level of 0.05.

## Results

Thirty patients of diverse ethnic backgrounds (21 female, 70%) with Paprosky type-3B defects, including 26 patients from the previous study, were followed for a median of 84 months (range, 65 to 109 months). The median age was 70 years (49 to 90 years). Pelvic discontinuity was confirmed intraoperatively for 4 (13%) of the patients (Table 1). Preoperative pelvic magnetic resonance imaging (MRI) was available for 14 (47%) of the patients and revealed mild to extensive fatty atrophy of the gluteal muscle complex with intact tendons in most cases; 1 patient demonstrated tendon detachment. Femoral stems were retained in 18 (60%) of the patients; others required cemented stems (6 patients), modular tapered stems (4 patients), or proximal femoral replacements (2 patients). Eleven (37%) of the patients underwent 2-stage revision for infection.

**Table 1:** Characteristics of the Cohort

No. of hips	30
Age *(yr)*	
Mean	69
Median	70
Range	49-90
Sex*	
Female	21 (70%)
Male	9 (30%)
Body mass index *(kg/m*^*2*^*)*	
Mean	28
Median	26
Range	22-42
Side*	
Right	18 (60%)
Left	12 (40%)
Hips with discontinuity*	4 (13%)
Clinical follow-up *(mo)*	
Mean	86
Median	84
Range	65-109

* Data are presented as the number of hips, with the percentage in parentheses.

CT imaging was performed for all patients immediately postoperatively and at 1 year postoperatively (30 of 30, 100%). Subsequent CT scans were obtained selectively on the basis of clinical suspicion of migration (year 2: 23 of 30 [77%]; year 3: 22 of 30 [73%]). Standard radiographs were obtained at all visits. OHS and mobility scores were recorded at baseline (OHS: 73% of the patients; mobility score: 100%) and the most recent follow-up (OHS: 70% of the patients; mobility score: 100%).

### Radiographic Outcomes

Postoperative imaging was evaluated for evidence of new bone formation, component integrity, and implant migration.

### Bone Formation and Component Integrity

**Fig. 2:**
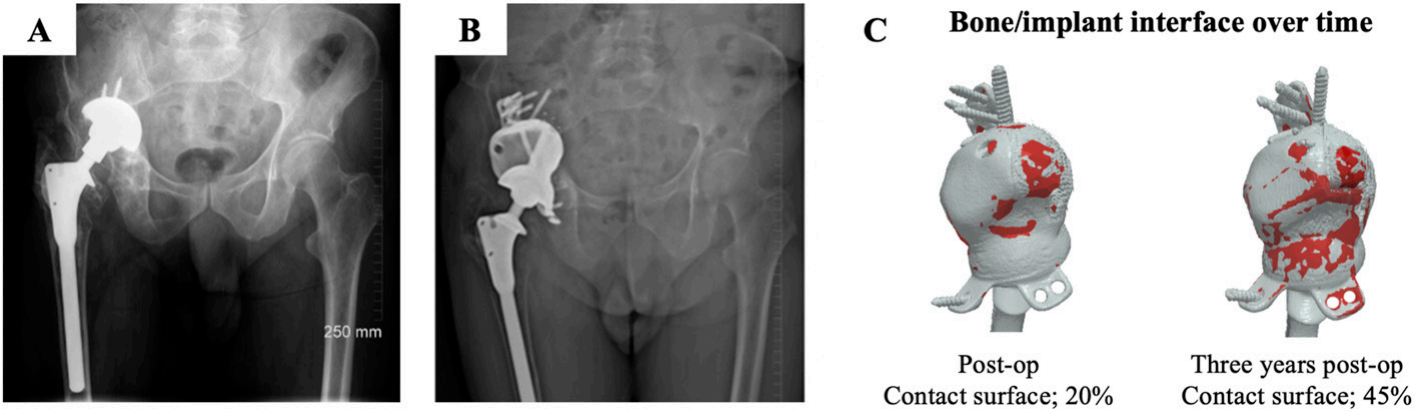
**Fig. 2-A:** Preoperative anteroposterior radiograph showing the failed implant on the patient’s right side. Medial and superior migration of the acetabular cup is observed. **Fig. 2-B:** Anteroposterior radiograph immediately postoperatively showing the custom implant in situ with the restored center of rotation. **Fig. 2-C:** Three-dimensional CT reconstructions of the implant immediately postoperatively and at 3 years postoperatively, illustrating an increase in the bone-implant contact areas (red regions) over time. The flanges and screws were excluded from the quantitative analysis; however, all screws were in bone.

Immediately postoperatively, bone-implant contact was found to be partial; on average, 15% of the implant was in contact with bone (range, 5% to 50%). Bone formation at the bone-implant interface was observed in 27 (90%) of the patients at ≥12 months of follow-up and was observed in areas of the acetabular wall and around the flanges (Fig. 2). Three patients exhibited no obvious signs of bone ingrowth on radiographs or CT.

No component breakage or major implant migration (>5 mm) was observed at ≥5 years, except in 1 patient. This patient developed hip pain 3 years postoperatively; SPECT-CT confirmed loosening. At the time of this report, the implant remains in situ under surveillance.

### Implant Migration

**Fig. 3:**
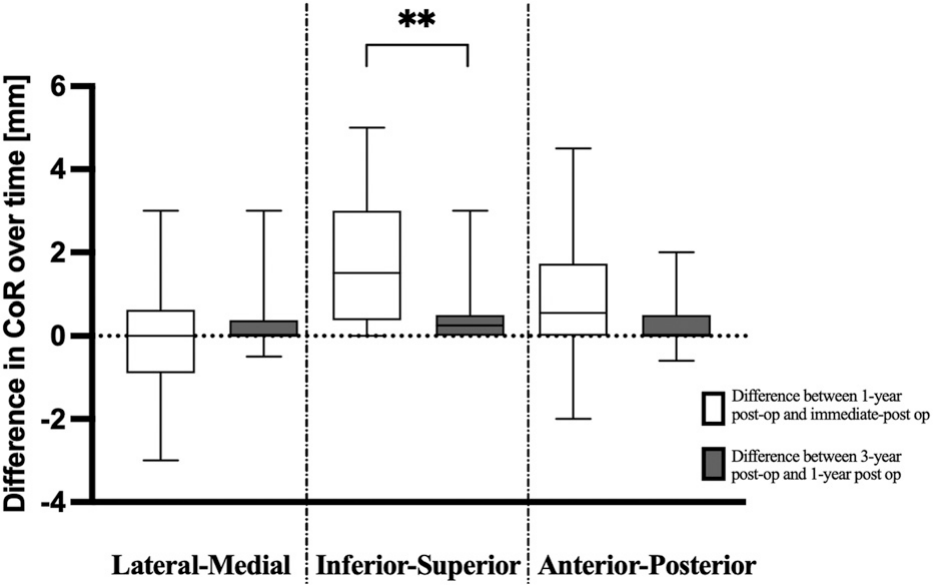
Box plots showing the difference in the center of rotation (CoR), in each direction, between the immediate postoperative and 1-year postoperative implant position (white boxes) and between the 1- and 3-year postoperative implant position (gray boxes). The boxes represent the interquartile range, the horizontal lines within the boxes represent the median values, and the whiskers represent the minimum and maximum values. **P = 0.0073.

At 1 year postoperatively, none of the implants had migrated >5 mm in any direction; the range was 0 to 5 mm, with 1 patient showing 5 mm of superior migration. Migration was <5 mm for all others (Fig. 3). The deviation of the CoR from immediately postoperatively to 1 year was a median of 0 mm (interquartile range [IQR], −1 to 0.6 mm) in the lateral-medial plane, 1.5 mm (IQR, 0.4 to 3 mm) in the inferior-superior plane, and 0.5 mm (IQR, 0 to 1.7 mm) in the anterior-posterior plane.

At 3 years postoperatively, the deviation of the CoR since 1 year postoperatively was a median of 0 mm (IQR, 0 to 0.4 mm) in the lateral-medial plane, 0.25 mm (IQR, 0 to 0.5 mm) in the inferior-superior plane, and 0 mm (IQR, 0 to 0.5 mm) in the anterior-posterior plane. None of the cups migrated >3 mm in any direction (Fig. 3). Implant migration was significantly reduced after the first year, particularly in the inferior-superior plane (p = 0.0073). Bone-to-bone registration provided a good match, with an average surface distance of 0.6 mm.

Beyond 3 years, both 2D and 3D analyses confirmed negligible additional migration, within the measurement error (<1 mm). Only 3 patients demonstrated minor residual migration, without radiographic evidence of loosening.

### Clinical Outcomes

#### Implant Survivorship

**Fig. 4:**
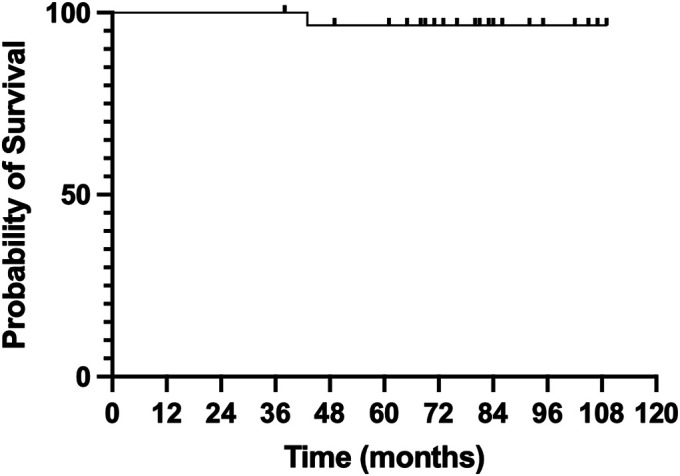
Kaplan-Meier survival curve for the custom-made 3D-printed titanium implants, with re-revision for any cause as the end point.

With clinical failure resulting from any cause as the end point, the overall Kaplan-Meier survival rate for the CMAI was 96.3%, with 26 patients at risk (Fig. 4).

There was 1 re-revision due to the recurrence of infection. The time from reconstruction with the custom implant to implant removal was 43 months; a cement spacer was inserted. No further surgery has occurred to date.

#### Functional Outcomes

The median OHS increased from 8 (IQR, 4 to 13; range, 2 to 21) preoperatively to 32 (IQR, 21 to 37; range, 14 to 41) at the previous, mid-term follow-up. The difference was significant (p < 0.0001). The score remained essentially unchanged (p > 0.5) at the most recent follow-up: 33 (IQR, 29 to 39; range, 14 to 46).

Mobility scores were recorded pre- and postoperatively, including at the most recent follow-up. Mobility scores improved significantly, by a mean of 1.4 points (range, −1.0 to +4.0; p < 0.0001), with 1 patient’s mobility having declined because of unrelated spinal and knee issues.

#### Complications

One patient experienced 2 dislocations, at 1 and 6 months, both managed with closed reduction; their OHS was 27 at 1 year, and their mobility score improved from 6 to 4. This patient died at 49 months postoperatively from unrelated causes. Another patient developed a transient sciatic nerve palsy with foot drop that resolved, and had an OHS of 30 and unaided walking at the time of the most recent follow-up. Overall, 3 patients died at 38, 49, and 61 months, for reasons unrelated to the hip. No new infections occurred, although 2 patients with longstanding infections had recurrence: 1 managed with suppressive antibiotics (implant retained), and the other undergoing re-revision at 43 months. One patient developed new-onset hip pain beyond 3 years; SPECT-CT confirmed cup loosening, and the patient remains under surveillance with the implant in situ. No fractures were observed.

#### Statistical Analysis

No correlation was found between the deviation of the CoR and BMI, age, sex, or pelvic discontinuity (p > 0.05).

## Discussion

Reliable mid- to long-term outcomes were demonstrated in a series of patients with severe acetabular bone loss who underwent reconstruction with a custom-made (enovis) implant between 2016 and 2020.

Several strategies exist for managing acetabular bone loss^4,13-15^. Over the past decade, clinical practice has increasingly favored uncemented reconstruction techniques, while the use of oblong cups, structural allografts, reconstruction rings, or cages has declined^16,17^. CMAIs have emerged as a promising alternative^1^. However, data remain limited to small series with relatively high complication (29% to 35%) and reoperation (15% to 19%) rates^18-22^.

CT-based implant measurement offers precise monitoring without bone markers, potentially replacing radiostereometric analysis (RSA) in long-term surveillance^23-26^. We previously showed that CT data can be used to assess plan compliance by comparing the planned and achieved positioning and orientation^7^. Additionally, both our findings and those of other investigators indicate that bone remodeling may be observed in the first year after surgery, especially in the presence of pelvic discontinuity^27-29^.

To our knowledge, this is the largest series with mid- to long-term results. We observed that, since the implant design required extensive bone preparation to accommodate it, the postoperative implant-bone contact area was only partial^1,29^. Over time, imaging revealed an increased contact area, indicating subtle implant migration toward a more stable position within the first postoperative year. This progressive seating, accompanied by osseointegration, reflects adaptive migration of the implant as it conforms to the surrounding bone and achieves biological fixation.

Most patients demonstrated bone formation at the implant interface at 12 months, with stability maintained up to 9 years. Only 1 implant showed loosening on SPECT-CT and remains under observation. The implant survival rate was 96.3%, with 1 re-revision for infection.

We acknowledge limitations. First, the accuracy of the 3D analysis depends on successful CT registration, which can be affected by metal artifacts. This was mitigated using artifact reduction and evaluated using the average surface distance (ASD) between corresponding pelvic models, yielding satisfactory results. Analyses were performed by experienced engineers. Second, all surgeries were performed by a single, high-volume surgeon, limiting reproducibility across different surgeons or implant systems. Lastly, the small sample size, inherent to the rarity of such complex reconstructions, restricts generalizability.

### Conclusions

CMAIs provide durable fixation for Paprosky type-3B defects, with implant migration limited to the first postoperative year as part of an adaptive osseointegration process. Long-term follow-up demonstrated a high rate of survivorship, sustained functional improvement, and few complications. Advanced imaging remains a valuable adjunct for postoperative monitoring.
